# Is there a genetic cause for cancer cachexia? – a clinical validation study in 1797 patients

**DOI:** 10.1038/bjc.2011.323

**Published:** 2011-09-20

**Authors:** T S Solheim, P M Fayers, T Fladvad, B Tan, F Skorpen, K Fearon, V E Baracos, P Klepstad, F Strasser, S Kaasa

**Affiliations:** 1 Department of Cancer Research and Molecular Medicine, Faculty of Medicine, Norwegian University of Science and Technology (NTNU), Trondheim 7030, Norway; 2 Department of Oncology, St Olavs University Hospital, Olav Kyrres gt 13, Trondheim 7006, Norway; 3 Department of Public Health, University of Aberdeen, Foresterhill, Aberdeen AB25 2ZD, UK; 4 Department of Laboratory Medicine, Children's and Women's Health, Faculty of Medicine, Norwegian University of Science and Technology (NTNU), Trondheim 7030, Norway; 5 Department of Clinical Surgery, University of Edinburgh, Clinical and Surgical Sciences (Surgery), Royal Infirmary, 51 Little France Crescent, Edinburgh EH16 4SA, UK; 6 Department of Oncology (Division of Palliative Care Medicine), University of Alberta, Edmonton, Alberta, Canada T6G 1Z2; 7 Department of Anaesthesiology and Emergency Medicine, St Olav University Hospital, Trondheim 7006, Norway; 8 Department of Circulation and Medical Imaging, Norwegian University of Science and Technology, Trondheim, Norway; 9 Oncological Palliative Medicine, Division of Oncology, Department of Internal Medicine and Palliative Care Center, Cantonal Hospital, St Gallen, Switzerland

**Keywords:** cachexia, polymorphism, validation, genetic

## Abstract

**Background:**

Cachexia has major impact on cancer patients’ morbidity and mortality. Future development of cachexia treatment needs methods for early identification of patients at risk. The aim of the study was to validate nine single-nucleotide polymorphisms (SNPs) previously associated with cachexia, and to explore 182 other candidate SNPs with the potential to be involved in the pathophysiology.

**Method:**

A total of 1797 cancer patients, classified as either having severe cachexia, mild cachexia or no cachexia, were genotyped.

**Results:**

After allowing for multiple testing, there was no statistically significant association between any of the SNPs analysed and the cachexia groups. However, consistent with prior reports, two SNPs from the acylpeptide hydrolase (APEH) gene showed suggestive statistical significance (*P*=0.02; OR, 0.78).

**Conclusion:**

This study failed to detect any significant association between any of the SNPs analysed and cachexia; although two SNPs from the *APEH* gene had a trend towards significance. The *APEH* gene encodes the enzyme APEH, postulated to be important in the endpoint of the ubiquitin system and thus the breakdown of proteins into free amino acids. In cachexia, there is an extensive breakdown of muscle proteins and an increase in the production of acute phase proteins in the liver.

Cachexia is characterised by anorexia, fatigue, weight loss, decreased muscle mass and inflammation (Evans *et al*, 2008), and has major effects on mortality and morbidity in cancer patients. One-third of all cancer patients lose >50% body weight, and it is estimated that cachexia accounts for 20% of cancer-related deaths, presumably largely due to cardiac or respiratory failure or complications from immobility (Fearon, 2008).

The aetiology of cancer cachexia is largely unknown. Furthermore, there is no universally accepted and validated definition of cachexia for use in clinical practice. Weight loss and low body mass index (BMI) have been considered the main indicators, but criteria that identify patients with both adverse function and prognosis are often integrated in the definitions (Fearon *et al*, 2006; Evans *et al*, 2008; Bozzetti and Mariani, 2009).

The lack of an accepted and validated definition has been a limitation in both genetic and clinical cachexia research. Recently, research groups have endeavoured to establish diagnostic criteria (Fearon *et al*, 2006, 2011; Evans *et al*, 2008; Bozzetti and Mariani, 2009), resulting in a consensus article (Fearon *et al*, 2011) with a proposed framework for the definition and classification of the condition. After validation, this could aid both pathogenetical research and clinical trial design, and facilitate the development of practice guidelines.

At present it cannot be predicted which patients will develop cachexia (Tan *et al*, 2008), but research into the treatment of cachexia demands methods for early identification of these patients (Muscaritoli *et al*, 2010). In seeking predictors either to guide management of the condition or to identify patients at risk of developing severe cachexia, one may consider patient characteristics, tumour biology or biochemical parameters as potential markers (Tan *et al*, 2008).

The seemingly unpredictable occurrence of cachexia is the foundation for the theory that there may be genetic predisposition to cachexia. To date, only a few studies have addressed the association between genetic markers in candidate genes and cachexia (Jatoi *et al*, 2007; Zhang *et al*, 2008; Deans *et al*, 2009). However, a majority of these studies are small and the numbers of polymorphisms investigated has been limited. Studies have found associations with cachexia and polymorphisms in IL-1-*β* (Jatoi *et al*, 2007; Zhang *et al*, 2007), IL-10 (Deans *et al*, 2009), IL-6 (Zhang *et al*, 2008) and IL-8 (Bo *et al*, 2010) genes. A IL-10 polymorphism (rs 1800896) is observed to be associated with cachexia in two studies; first with a >10% weight loss in a Scottish gastro-oesophageal cancer population (Deans *et al*, 2009), and recently in a Chinese gastric cancer population (Sun *et al*, 2010). The IL-1-*β* polymorphism (rs1143634) has, however, been reported to be associated both with weight loss (Zhang *et al*, 2007) and with greater improvement in weight in a prospective study (Jatoi *et al*, 2007) (Table 1). For all SNPs, there is a lack of validation studies with large sample sizes in heterogeneous cancer populations.

The possible genetic contribution to cachexia is presumably complex and there may be multiple genetic and environmental components contributing to susceptibility for the condition (Hirschhorn *et al*, 2002). This complex pathophysiology adds to the methodological challenges commonly encountered when studying genetic associations. False-positive associations are common because of multiple statistical testing, while true positives are few because of low power from inadequate sample sizes (Garner, 2007). A review of publications on genetic associations with common diseases found that only 4% of the reported associations could be confirmed in later studies (Hirschhorn *et al*, 2002). Other explanations for not being able to confirm genetic associations may be variable linkage disequilibrium between the polymorphism studied and the causal variant, the ethnic mixture or the gene–gene/gene–environment relations specific for the studied populations (Hirschhorn *et al*, 2002). Moreover, variations in DNA do not always lead to changes in proteins, and altered protein constitutions are not necessarily accompanied by any clinical significance. Thus, there is a need for thorough investigations and confirmatory studies to establish the impact of polymorphisms.

The primary research question in this study is: can the association between cachexia and any of the nine previously reported single-nucleotide polymorphisms’ (SNPs) be confirmed? The two secondary research questions are: (1) is it possible to generate new hypothesis on the pathophysiology by considering the associations with 191 new candidate SNPs and cachexia? and (2) is the definition established in this study consistent with known characteristics associated with cachexia?

## Patients and methods

### Patients and study design

Between February 2004 and April 2008, 2312 cancer patients were included in the European Pharmacogenetic Opioid Study (EPOS) (Klepstad *et al*, 2011). This was a multi-centre, cross-sectional, observational study, which included patients with cancer at different sites, stages and with different performance status who received opioid treatment. Patients were recruited at 17 centres in 11 different countries. Patients aged <18 years or not capable of understanding the language used at the study centre were not eligible.

The appropriate ethical authorities in all participating centres approved the study protocol, and all patients gave their oral and written informed consent.

### Clinical assessment

Age, ethnicity, gender, weight, height, Karnofsky performance status and medication during the last 24 h were recorded. Cancer diagnosis, presence of metastases and time since the diagnosis of cancer were registered. Body mass index (BMI) was evaluated according to the WHO scale. Information on survival was collected until January 2010. Patients’ subjective health at the time of inclusion was measured by the European Organisation for Research and Treatment of Cancer Core Quality of Life Questionnaire, the EORTC QLQ-C30 version 3.0 (Aaronson *et al*, 1993). In this questionnaire, the patients report symptoms for the past week on a four-point verbal rating scale: (1) not at all, (2) a little, (3) quite a bit and (4) very much.

### The cachexia phenotype

In order to classify cachexia, the following dichotomised factors were applied:

- BMI: <20 kg m^–2^
- Karnofsky score: <80
- CRP: ⩾10 mg l^–1^
- Appetite loss: a response of little or greater on EORTC QLQ-C30 item ‘have you lacked appetite?’

Patients were divided into three groups dependent on whether they had all four cachexia factors (severe cachexia), two or three cachexia factors (mild cachexia) or less than two cachexia factors (no cachexia).

### Blood sampling

Blood samples were collected at the time of inclusion and stored at −80 °C before shipment to the Norwegian University of Science and Technology (NTNU) in Trondheim, Norway for DNA extraction and CRP analysis.

#### Polymorphisms for validation analysis.

Literature published before 2009 was reviewed before genotyping. This identified two polymorphisms that had been reported to be associated with cancer cachexia (Jatoi *et al*, 2007; Zhang *et al*, 2007; Deans *et al*, 2009). In addition, a study performed in parallel with this study as a part of our group's research on genetics and cachexia identified seven SNPs to be associated with cachexia (personal communication Tan). These seven SNPs are based on the preliminary analyses using a permutation test with *P*<0.01 to indicate significance. Thus, a total of nine SNPs were selected to be included in the present validation analysis. The polymorphisms to be validated are presented in Table 1.

#### Polymorphisms for exploratory analysis.

Before the genetic analyses, a further 182 candidate SNPs from 99 candidate genes were identified as having a putative impact on cachexia pathophysiology (see Supplementary Table 1). These genes code for pro-inflammatory cytokines, anti-inflammatory cytokines, endocrine hormones, muscle signalling pathways, muscle protein degradation pathways and appetite regulation. In addition, 18 candidate SNPs were chosen based on the results from gene expression array analysis of muscle samples from cancer patients with cachexia (Stephens *et al*, 2010).

#### Genotyping and serum analyses.

Genomic DNA extraction was performed at HUNT Biobank, Levanger, Norway. DNA was extracted from EDTA whole blood using the Gentra Puregene blood kit (QIAGEN Science, Germantown, MD, USA).

Genotyping was performed using the SNPlex Genotyping System (Applied Biosystems, Foster City, CA, USA) according to the suppliers’ dry DNA protocol. The capillary electrophoresis was carried out with an ABI 3730 48-capillary DNA analyser (Applied Biosystems). SNPlex signals were analysed using the Gene Mapper version 4.0 software (Applied Biosystems), followed by manual reading. Samples giving low signals, which could not be discriminated from the negative controls were removed before the analysis and treated as missing data. Genotype clustering was performed based on the SNPlex Rules 3730 method, following factory default settings. This method also includes quality control where a SNP has to exceed the 80 % call rate to pass. The software also rejects all sample wells where the well's behaviour deviates from the characterisation of an ideal well. Two SNPs, rs4680 and rs1045642, which could not be analysed by the SNPlex system, were genotyped at the HUNT Biobank. They were analysed by using TaqMan SNP allelic discrimination by means of an ABI 7900HT. All genotyping procedures were processed without regard of the phenotype (all genotyping processes were performed according to Applied Biosystems).

Haemoglobin, albumin and CRP were measured by standard clinical chemistry analytical methods.

### Statistical analyses

Demographic- and disease-related factors were explored for possible association with the cachexia groups. The factors explored were age, gender, cancer site, time since diagnosis and presence of metastasis. Variables such as Karnofsky status were not included as potential explanatory factors because they may also be affected by cachexia.

Factors were explored for association with cachexia using ordinal logistic regression and those that were significant (*P*<0.05) in univariate analyses were subsequently included in a stepwise multivariate regression stratified by country. Significant (*P*<0.05) factors from the multivariate analyses were included as covariates in the genetic analyses.

The association between SNPs and cachexia was analysed by ordinal logistic regression with cachexia groups as the outcome variable. All regression analysis was stratified by country.

Analyses were also repeated without the inclusion of covariates, as a sensitivity check. Before exploring the genetic associations, SNPs were rejected if the genotypes were not in Hardy–Weinberg equilibrium (HWE) (*χ*^2^-test, *P*<0.0005) or had minor allele frequencies (MAF) <5%. In the exploratory analysis of the 182 candidate SNPs, the patients were randomly divided into two groups in the ratio 1 : 2. The largest group with 2 out of 3 of the patients was used as a development sample, and the smaller group for confirmation of significant results found in the development sample. For the validation analyses of the nine SNPs identified in previous studies, the initial exploratory analysis was deemed unnecessary, and the entire sample of patients was used in the association analysis.

Three approaches were adopted to mitigate the multiplicity issues. First, a false discovery rate (FDR) of 10% was used for reporting the Benjamini–Hochberg (B–H) thresholds (Benjamini and Hochberg, 1995). If a SNP is to be classified ‘significant’, its *P*-value has to exceed the B–H criterion. Second, the co-dominant genetic model was pre-specified for the primary analyses, with other models (dominant, recessive, additive) being considered as exploratory analyses. A recessive trait will only be expressed if the dominant allele is not present. In a co-dominant model both alleles are visible in the phenotype, and in an additive model the two alleles have an enhancing effect on each other's influence on the phenotype. Third, interpretation of *P*-values was done with caution. STATA version 11.0 was used for all analyses (StataCorp LP, College Station, TX, USA, 2009).

The characteristics of the three cachexia groups were compared using ANOVA for the continuous data and *χ*^2^ for the categorical data. CRP was not normally distributed and was analysed using the non-parametric Kruskal–Wallis test. Survival and time since diagnosis was analysed by log-rank test.

## Results

**Figure 1:**
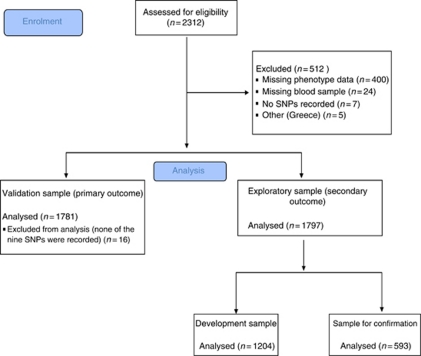
Flowchart demonstrating enrolment and analysis samples.

A total of 1797 patients with complete data on BMI, CRP, Karnofsky performance score, genetic analyses and appetite score were available for analyses after excluding five patients from Greece because of their low number, 61 patients not of European descent, 400 patients because of incomplete phenotyping data, 24 patients because of missing blood samples and 7 patients because of no SNPs recorded (see Figure 1). Out of the 1797 patients left for analyses, 194 patients had all 4 cachexia factors (severe cachexia), 1304 patients had two or three cachexia factors (mild cachexia) whereas 299 patients had less than two cachexia factors (no cachexia).

After applying first univariate and then multivariate ordered regression analysis of covariates potentially associated with cachexia, 11 variables were considered to be of prognostic importance for grade of cachexia. Gender, time since diagnosis, bone metastasis and several cancer diagnoses were not significantly associated and were therefore omitted in the subsequent genetic analyses (see Table 2).

### Validation sample

Nine candidate SNPs shown in previous studies to be associated with cachexia were assessed (Table 1). All SNPs had a MAF >5%, and genotype frequencies did not deviate from the HWE. Sixteen patients failed genotyping, leaving 1781 patients for final analysis.

No SNPs were significantly associated with cachexia, neither in the co-dominant, dominant, additive nor recessive model. Table 3 presents the results of the co-dominant model. The *acylpeptide hydrolase* (*APEH*) SNP rs 4855881 showed a trend toward significance (*P*=0.0199), but the *P*-value was still above the B–H criterion of 0.011 that we pre-specified as denoting significance.

### Exploratory sample

The 182 candidate SNPs were analysed in order to identify new associations between cachexia and genetic polymorphisms. After 25 SNPs were excluded because of violation of HWE or for having a MAF <5%, 157 candidate SNPs remained.

The development and confirmatory sample were well balanced for age, BMI, Karnofsky performance status and the three cachexia groups (data not shown).

No SNPs were significantly associated with cachexia in both the development sample and the confirmatory sample, neither in the co-dominant, dominant, additive nor recessive model. The five most significant SNPs in the dominant model are presented in Table 4. A SNP in *IRS1* (rs2234931) was significantly associated with cachexia in the exploratory analysis. (*P*=0.000045, B–H criterion=0.00061), but was not significant (*P*=0.29184) in the confirmatory sample, and the odds ratio also showed an effect in opposite direction from that in the development sample.

### Patient characteristics in cachexia patients and controls

**Figure 2:**
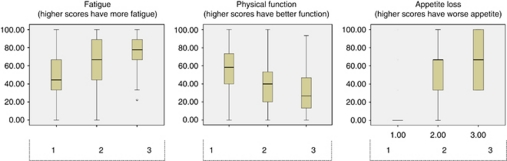
Box plots of QoL parameters with the three cachexia groups as the dependent variable. (1) None or one cachexia factors (no cachexia). (2) Two or three cachexia factors (mild cachexia). (3) All four cachexia factors (severe cachexia). Cachexia factors: BMI<20 kg m^–2^, appetite⩾33, CRP⩾10, Karnofsky<80. EORTC: European Organisation for Research and Treatment of Cancer Quality-of-Life Core Questionnaire (EORTC-QLQ-C30) version 3.0.

The general characteristics of the patients are presented in Table 5. Figure 2 presents QoL parameters distributed according to number of cachexia factors.

The group with severe cachexia had also significantly lower median survival of 54 days whereas the mild cachexia group had a survival of 103 days and the patients with no cachexia survived for a median 304 days after inclusion. There were significant differences in fatigue and appetite loss among the groups (Figure 2).

There was also significantly lower physical function and lower Karnofsky score between the groups dependent on where in the cachexia trajectory they were classified. CRP (*P*<0.0001), albumin (*P*<0.0001) and haemoglobin (*P*<0.0001) were also significantly different among the cachexia groups.

## Discussion

### Primary and secondary aims

None of the nine SNPs previously reported to be associated with cachexia were associated with cachexia in this study. However, two SNPs from the *APEH* gene showed a trend toward significance. The *APEH* gene encodes the enzyme APEH, which is postulated to be important in the endpoint of the ubiquitin proteasome system and thus the breakdown of proteins into free amino acids (Perrier *et al*, 2005). In cachexia, there is an extensive breakdown of muscle proteins and an increase in the production of acute phase proteins in the liver (Fearon, 2008), so this gene is of interest in cachexia pathophysiology. In the first study, where the *APEH* gene was described as significantly associated with the condition, cachexia was classified as a spectrum. The cut-offs were <5, <10 and <15% weight loss, with or without systemic inflammation. In all phenotypes with elevated CRP, *APEH* was significantly associated with weight loss (*P*<0,01), but not when the phenotype was weight loss without CRP elevation (personal communication Tan) The phenotype applied in this study also involves elevated CRP. This might imply that the effect of the enzyme APEH is important during inflammation in cachexia.

In this study, the total population of 1797 patients was divided in two groups when exploring the 157 new candidate SNPs. One SNP was significantly associated with cachexia in the development sample, but was not significant in the confirmation sample. Thus, no significant associations were discovered. There is a high risk of false-positive results in genetic association studies because of multiple testing and low power (Garner, 2007), and the lack of replication has been a major limitation (Pasche and Yi, 2010). Although a few genes have been validated, many reports of genetic associations in common diseases have proved irreproducible in large validation studies or meta-analysis.

### Third aim

#### Reasons for the definition.

Reliable phenotyping is a critical component in genetic association studies of complex diseases. This challenge particularly applies to cancer cachexia where there still is no universally accepted and validated definition, and the pathophysiology is presumably very complex. When different investigators propose multiple factors to define cachexia, each factor's ability to predict shorter survival is sometimes explored. In order to select a population with reduced survival, weight loss and symptoms associated with severe cachexia, we defined severe cachexia as BMI<20 kg m^–2^, anorexia, reduced Karnofsky performance status and concomitant systemic inflammation. In several classification systems, BMI<20 kg m^–2^ has been used to distinguish between the cachectic and non-cachectic patients (Evans *et al*, 2008; Fearon *et al*, 2011), and most patients with low BMI are sarcopenic (Antoun *et al*, 2010). Anorexia is often not considered mandatory in order to categorise a patient as cachectic, but the majority do suffer some loss of appetite (Sarhill *et al*, 2003). Karnofsky performance score is associated with weight loss (Blum *et al*, 2011) and is one of the most powerful predictors for survival in cancer (Maltoni *et al*, 2005). Lower Karnofsky score will also incorporate the impact of cachexia on physical function. Both weight loss and the adverse effects of cachexia such as fatigue and performance status have been documented associated with systemic inflammatory response (Scott *et al*, 2002) and CRP has in several studies and reviews proved to be an independent marker for survival (Maltoni *et al*, 2005). The most robust biomarker for cachexia today is therefore probably elevated CRP (Tan *et al*, 2008) and it is thus included in the current four-factor definition.

#### Validity of the definition.

A third aim of this study was to see whether the four-factor profile for refractory cachexia was in coherence with known clinical characteristics of the condition. When taking into consideration the multidimensional aspects of cachexia, the prevalence will be lower than if applying weight loss as the sole criterion. A previous study reported that over 80% of pancreatic cancer patients had >10% weight loss, while only 20% of the same patients fulfilled a three factor profile of cachexia (Fearon *et al*, 2006). This three factor profile, however, proved to embrace patients with both reduced survival and function. Cancer cachexia is a spectrum ranging from mild cachexia to severe cachexia. A stringent cachexia definition was attempted applied in this study in order to identify patients with severe cachexia. As could be expected, there are rather few patients at this end of the spectrum. In all, 11% of the included study patients had all four cachexia factors, but the frequency of severe cachexia varied with type of cancer (Table 3). This is in accordance with previous studies (Bozzetti and Mariani, 2009). The three cachexia groups that were defined had also gradually higher CRP, lower albumin and lower haemoglobin levels. These alterations in biochemical markers have previously been suggested as diagnostic criteria for the cachexia (Evans *et al*, 2008).

Not all cancer patients who lose weight will develop severe cachexia, but it is important to identify patients at risk in an early phase if preventive measures or treatment is to succeed. However, the screening of patients to undergo preventive interventions requires markers with low false-positive rate and high positive predictive values to avoid unnecessary psychological or physical toll. Patients were found to have slightly more morbidity, worse biochemical parameters and lower survival according to whether they have no cachexia, mild cachexia or severe cachexia. It was not possible in this cross-sectional study to verify whether the patients with more cachexia factors had more weight loss.

### Evaluations of the aims

This study is the most comprehensive to date exploring genetic polymorphisms associated with cachexia. A heterogeneous cohort of cancer patients from different European countries was investigated in order to identify a polymorphism with high external validity. A majority of previous studies have looked at polymorphisms in the host within limited cancer diagnoses (Jatoi *et al*, 2007; Zhang *et al*, 2007; Deans *et al*, 2009; Bo *et al*, 2010). Cachexia is present in most cancer types, but prevalence and severity varies. One could speculate that if patients are prone to or protected from cachexia because of their genetic build-up, it would be the same genes involved independent of cancer diagnosis. However, there is no evidence to confirm this. The cachexia pathophysiology and the hosts’ reaction to the tumour might prove to be different depending on where the tumour originated.

Although we failed both to confirm previously published genetic associations, and to identify new possible genetic associations, our findings do not allow for ruling out genetics in cancer cachexia pathophysiology. Although the selection of genes in this study has a high theoretic rationale, the complexity of the human genome suggests there may well be other genes and SNPs involved in cachexia. Also the genetics of the tumour are likely to influence cancer cachexia. Moreover, a Chinese research group recently published associations with cachexia and SNPs in the IL-6 (Zhang *et al*, 2008), IL-8 (Song *et al*, 2009) and IL-10 (Sun *et al*, 2010) genes. As these associations were published after the SNPs in this study were genotyped, they were thus not available to us for validation. In this study, only single SNPs were explored and not multi-SNP interactions because of the relatively limited sample size.

In addition to exploring 157 new candidate SNPs, this study is the first performed with the primary aim to verify polymorphisms previously reported associated with cachexia. If the genetic effect of these nine SNPs is weak, their association with cachexia might still be true, although not significantly reproduced in the present cohort of cancer patients. Owing to the variation in samples of patients, each effect size that is reported is unavoidably imprecise. The effect of an association tends to be over – rather than underestimated by the first group that reports it (Hirschhorn *et al*, 2002), and a weak effect may not be detectable in a small under-powered sample. To establish or confirm a polymorphism with large external validity, but with weak effect size, it is necessary to design larger studies or use meta-analysis.

A polymorphism with a weak effect size or which is present only in a restricted population may still be important clinically. Such a polymorphism might give information on pathophysiology, and may aid in developing new drugs as cachexia is at present largely untreatable (Hirschhorn *et al*, 2002). In this study, only two of nine candidate SNPs showed associations, albeit not statistically significant after allowing for multiple testing. Both of these SNPs are from the *APEH* gene. Based on the theory of weak genetic effects, it would be of interest to do further investigations on the effect of APEH in cachexia.

A challenge in association studies is that the effect size of the SNPs previously published may be too small to apply to populations moderately different from those studied. Some studies looking at polymorphisms and cachexia have chosen >10% weight loss as a phenotype (Deans *et al*, 2009). The phenotypes might not be directly comparable since the fraction of patients with weight loss will be much larger than the fraction of patients who suffer from severe cachexia with its consequences on morbidity and mortality (Fearon *et al*, 2006). The present phenotype is a compound of factors that seems to describe the condition well, but nevertheless does not say anything about the underlying pathophysiology. Both weight loss and the present cachexia phenotype are probably too unspecific, and both include patients with very different pathophysiological paths, which lead to common endpoints. Cachexia is a complex condition, and a phenotype that attempts to include only patients that represent a single pathophysiological path, such as sarcopenia or well-described inflammation, may be more successful in discovering disease contributing genes.

In summary, the objective of this study was to validate nine SNPs previously described associated with cachexia and to explore 157 other candidate SNPs with the potential to be involved in the development of the condition. This study failed to detect any significant association with any of the SNPs analysed and cachexia, although two SNPs from the *APEH* gene had a trend towards significance. Further investigations are needed in order to establish the role of *APEH* in cancer cachexia.

## Figures and Tables

**Table 1:** Polymorphism attempted validated in the present study

	**Gene**	**RS-number**	***P*-values**	**Risk allele**	**Patients included**	**Phenotype**
Tan *et al* (2008)	SELP (selectin)	rs6136	0.000492	C	775 Patients with cancer at different sites and stages	6 Phenotypes
	LEPR (leptin receptor)	rs1137100	0.00821	G		(1) >5% Weight loss
	GHRL (ghrelin)	rs42451	0.006363	T		(2) >10% Weight loss
	TNFRSF1A (tumour necrosis factor receptor)	rs4149570	0.008415	T		(3) >15% Weight loss
	APEH (*N*-acylaminoacyl-peptide hydrolase)	rs4855881	0.000854	C		(4–6) The above with CRP concentration of >10 mg l^–1^
	APEH (*N*-acylaminoacyl-peptide hydrolase)	rs2960548	0.00298	G		
	Postfox01a (Forkhead box, sub-group O)	rs17446593	0.004702	G		
		−129 SNPs were investigated				
						
Deans *et al* (2009)	IL-10 (−1082)	rs1800896	0.014	G	203 Patients with gastro-oesophagal cancer	Weight loss >10% (80 patients)
		−5 SNPs were investigated	0.019			
						
Zhang *et al* (2007)	IL-1*β* (+3954)	rs1143634	0.018	T	214 Patients with locally advanced gastric cancer	Weight loss > 10% (91 patients)
		−4 SNPs were investigated				
						
Jatoi *et al* (2007)	IL-1*β* (+3954)	rs1143634−4 SNPs were investigated	0.02	T	44 Patients with metastatic gastric and gastro-oesophagal cancer	Phenotype is greater improvements in weight registered every 3 week during chemotherapy.

Abbreviations: APEH=acylpeptide hydrolase; CRP=C-reactive protein; IL=interleukin; SNP=single-nucleotide polymorphism.

**Table 2:** Covariates retained in the genetic association analysis

**Cachexia**	**Odds ratio**	***P*-value**	**95% Confidence interval**
Lung cancer	1.8	<0.001	1.4, 2.4
Skin cancer	2.7	0.001	1.5, 4.8
Head and neck cancer	2.7	0.000	1.6, 4.6
Pancreas cancer	5.0	0.000	2.6, 9.5
Sarcoma	2.2	0.026	1.1, 4.5
Gastrointestinal cancer	2.1	<0.001	1.5, 2.8
Female reproductive cancer	1.9	0.002	1.3, 2.8
Age	1.0	0.078	1.0, 1.0
Liver metastasis	1.3	0.055	1.0, 1.7
Other metastasis	1.7	<0.001	1.3, 2.1
Lung metastasis	1.5	0.004	1.1, 1.9

Dependent variable is three cachexia groups, based on number of cachexia factors. (1–0, 2–3, 4).

The *P*-values and 95% confidence interval (CI) are from multivariate analysis.

**Table 3:** Co-dominant model of the nine validation sample

**SNP**	***P*-value (*n*=1781)**	**B–H criterion**	**OR**
*APEH*			
rs4855881	0.0199	0.011	0.78
			
*APEH*			
rs2960548	0.0317	0.022	0.79
			
*Postfox01a*			
rs1744659	0.1368	0.033	1.19
			
*IL-1*β			
rs1143634	0.2180	0.044	0.87
			
*SELP*			
rs6136	0.5000	0.056	1.10
			
*TNFrsf1a*			
rs4149570	0.7933	0.067	1.03
			
*LEPR*			
rs1137100	0.8343	0.078	1.02
			
*IL-10*			
rs1800896	0.9005	0.089	1.01
			
*GHRL*			
rs42451	0.9426	0.100	1.01

Abbreviations: APEH=acylpeptide hydrolase; B–H= Benjamini–Hochberg; IL=interleukin; OR=odds ratio; SNP=single-nucleotide polymorphism; TNF=tumour necrosis factor.

**Table 4:** Dominant model, the five most significant SNPs

**SNP**	***P*-value; development sample (*n*=1204)**	**OR**	**B–H criterion**	***P*-value; sample for confirmation (*n*=593)**	**OR**
*IRS1 (insulin receptor substrate)* rs2234931	0.000045	1.88	0.00061	0.29184	0.44
					
*LITAF (lipolysaccaride-induced tumour necrosis factor*-*α)* rs4280262	0.013427	0.70	0.00123	0.00099	1.92
					
*postfox01a (Forkhead box, sub-group O)* rs1744659	0.024845	1.37	0.00184	0.95283	0.99
					
*TTC18 (tetratricopeptide repeat domain 18)* rs3812621	0.026004	0.65	0.00245	0.91230	1.03
					
*IL-12β* rs1368439	0.031608	2.12	0.00306	0.48657	1.26

Abbreviations: B–H=Benjamini–Hochberg; IL=interleukin; OR=odds ratio; SNP=single-nucleotide polymorphism.

**Table 5:** Characteristics of the three cachexia groups

	**0–1 Cachexia factors (*n*=299)**		**2–3 Cachexia factors (*n*=1304)**		**4 Cachexia factors (*n*=194)**		***P*-values**
*Gender*							
Male	153	51%	688	53%	88	45%	0.143
Female	146	49%	616	47%	106	55%	
							
Age	61 (25–88)		63 (18–91)		62 (19–91)		0.589
Body mass index	25 (17–45)		24 (14–45)		19 (9–20)		<0.0001
							
*Department*							
Hospitalised	178	59%	1056	81%	170	88%	<0.0001
Outpatients	121	41%	248	19%	24	12%	
							
Survival in days (95% CI)	304 (246–362)		103 (93–114)		54 (40–68)		<0.0001
Time since diagnosis in months (95% CI)	25 (18–31)		14 (12–16)		15 (12–18)		<0.0001
							
Karnofsky performance status	80 (20–100)		60 (20–100)		50 (20–70)		<0.0001
							
*Tumour diagnosis*							
Urologic	20	7%	91	7%	14	7%	
Lung	35	12%	239	18%	32	17%	
Breast	67	22%	170	13%	21	11%	
Prostate	52	17%	145	11%	11	6%	
Gastrointestinal	44	15%	272	21%	54	28%	
Pancreas	0	0%	26	2%	9	5%	
Female reproductive organs	14	5%	109	8%	16	8%	
Head and neck	13	4%	70	5%	17	9%	
Haematological	21	7%	67	5%	6	3%	
Unknown origin	10	3%	36	3%	4	2%	
Sarcoma	6	2%	35	3%	7	4%	
Skin	1	0%	30	2%	5	3%	
Others	25	8%	66	5%	8	4%	
							
*Metastasis* a							
Liver	59	20%	319	24%	65	34%	
Bone	163	55%	589	45%	68	35%	
CNS	15	5%	70	5%	14	7%	
Lung	49	16%	290	22%	59	30%	
Other	88	29%	520	40%	105	54%	
None	56	19%	211	16%	27	14%	
							
*Medication last 24 h*							
Chemotherapy	61	20%	186	14%	24	13%	0.023
Steroids	145	49%	634	49%	887	46%	0.764
Antibiotics	44	15%	290	22%	40	21%	0.011
							
*Biochemical parameters (mean and 95% CI)*							
Hb (*μ* mol l^–1^)	11.9 (11.7–12.1)		11.4 (11.3–11.5)		10.8 (10.6–11.1)		<0.0001
Albumin (g l^–1^)	36 (35–37)		32 (31–32)		29 (28–30)		<0.0001
CRP (g l^–1^)	12 (8–16)		58 (55–61)		78 (67–86)		<0.0001

Abbreviations: CI=confidence interval; CNS=central nervous system; CRP=C-reactive protein; Hb=haemoglobin.

All numbers are absolute numbers or medians (range) if nothing else is indicated.

a Many patients can have more than one metastasis site.

## APPENDIX

**EPOS**

Principal investigator: Pål Klepstad, Trondheim, Norway. Investigator at each centre: Andrew Davies, Sutton/Chelsea, UK. Augusto Caraceni, Alessandra Pigni, Milan, Italy. Danilo Miotti, Pavia, Italy. Eeva Salminen, Turku, Finland. Eriphili Argyra, Athens, Greece. Florian Strasser, St Gallen Switzerland. Irena Poviloniene, Vilnius, Lithuania. Jon Håvard Loge, Oslo, Norway. Kristin Bjordal, Oslo, Norway. Lucas Radbruch, Aachen, Germany. Marianne Kloke, Essen Germany. Marco Maltoni, Forli, Italy. Per Sjøgren, Copenhagen Denmark. Rainer Sabatowski, Dresden, Germany. Staffan Lundström, Stockholm, Sweden. Stein Kaasa, Trondheim, Norway. Valgerdur Sigurdardottir, Reykjavik, Iceland.

**EPCRC**

The European Palliative Care Research Collaborative is funded by the European Commission's Sixth Framework Programme (contract no LSHC-CT-2006-037777) with the overall aim to improve treatment of pain, depression and fatigue through translation research. *Core scientific group*/*work package leaders:* Stein Kaasa (project coordinator), Frank Skorpen, Marianne Jensen Hjermstad, and Jon Håvard Loge, Norwegian University of Science and Technology (NTNU); Geoffrey Hanks, University of Bristol; Augusto Caraceni and Franco De Conno, Fondazione IRCCS Istituto Nazionale dei Tumori, Milan; Irene Higginson, King's College London; Florian Strasser, Cantonal Hospital St Gallen; Lukas Radbruch, RWTH Aachen University; Kenneth Fearon, University of Edinburgh; Hellmut Samonigg, Medical University of Graz; Ketil Bø, Trollhetta AS, Norway; Irene Rech-Weichselbraun, Bender MedSystems GmbH, Austria; Odd Erik Gundersen, Verdande Technology AS, Norway. *Scientific advisory group:* Neil Aaronson, The Netherlands Cancer Institute; Vickie Baracos and Robin Fainsinger, University of Alberta; Patrick C Stone, St George's University of London; Mari Lloyd-Williams, University of Liverpool. *Project management:* Stein Kaasa, Ola Dale, and Dagny F Haugen, NTNU.
