# Supplementary material for: Is there a genetic cause for cancer cachexia? – a clinical validation study in 1797 patients
Source: Br J Cancer. 2011 Sep 20;105(8):1244–51. doi: 10.1038/bjc.2011.323 (PMC3208484; doi:10.1038/bjc.2011.323)
Supplement: Supplementary Table 1 [file bjc2011323x1.doc]

**Table S1:** Details of SNPS analysed

| CHR | GENE | SNP | BP | A1 | A2 | HWE | MAF % |
| --- | --- | --- | --- | --- | --- | --- | --- |
| 1 | TNFRSF1B | rs496888 | 12155392 | G | A | 0.0081 | 28.2 |
| 1 | TNFRSF1B | rs976881 | 12156340 | A | G | 0.9606 | 33.4 |
| 1 | TNFRSF1B | rs1061622 | 12252955 | G | T | 0.9753 | 23.8 |
| 1 | TNFRSF1B | rs3397 | 12189878 | C | T | 0.5970 | 35.5 |
| 1 | TNFRSF1B | rs1061631 | 12191085 | A | G | 0.7693 | 19.9 |
| 1 | TAF12 | rs3795845 | 28804211 | C | A | 0.7684 | 28.1 |
| 1 | TAF12 | rs1804642 | 28821087 |  | G | 0 | 0 |
| 1 | DIO1 | rs11206244 | 54148288 | T | C | 0.9140 | 34.1 |
| 1 | DIO1 | rs11206246 | 54150613 | C | T | 0.6989 | 10.1 |
| 1 | LEPR | rs1137100 | 65809028 | G | A | 0.4031 | 26.1 |
| 1 | LEPR | rs1137101 | 65831100 | G | A | 0.6627 | 47.2 |
| 1 | LEPR | rs1805134 | 65839696 | C | T | 0.9482 | 22.3 |
| 1 | LEPR | rs8179183 | 65848539 | C | G | 0.7564 | 17.3 |
| 1 | VCAM1 | rs3176860 | 100959806 | G | A | 0.8939 | 40.9 |
| 1 | CRP | rs2794520 | 157945439 | T | C | 0.3109 | 32.1 |
| 1 | CRP | rs1130864 | 157949714 | T | C | 0.9056 | 32.3 |
| 1 | CRP | rs1800947 | 157950061 | C | G | 0.1476 | 6.2 |
| 1 | SELP | rs6136 | 167830574 | C | A | 0.7026 | 10.0 |
| 1 | IL10 | rs1800872 | 205013029 | A | C | 0.8416 | 23.5 |
| 1 | IL10 | rs1800896 | 205013519 | G | A | 0.0019 | 46.9 |
| 1 | HSD11B1 | rs12086634 | 207946881 | G | T | 0.7363 | 19.3 |
| 1 | HSD11B1 | rs2236903 | 207950102 | T | A | 0.6011 | 22.3 |
| 1 | TGFB2 | rs947712 | 216631503 | A | G | 0.9234 | 36.9 |
| 1 | TGFB2 | rs1890995 | 216671300 | T | C | 0.1317 | 27.5 |
| 1 | TGFB2 | rs1418553 | 216676876 | T | C | 0.2740 | 29.2 |
| 1 | TLR5 | rs5744168 | 221351822 | T | C | 0.2117 | 5.1 |
| 2 | LTBP1 | rs817529 | 33350838 | G | A | 0.4294 | 41.5 |
| 2 | IL1R1 | rs2228139 | 102148080 | G | C | 0.3785 | 6.3 |
| 2 | IL1A | rs17561 | 113253693 | T | G | 0.7919 | 29.8 |
| 2 | IL1A | rs1800587 | 113259430 | T | C | 0.9298 | 29.8 |
| 2 | IL1B | rs1143634 | 113306860 | T | C | 0.7264 | 25.1 |
| 2 | IL1B | rs1143627 | 113310857 | C | T | 0.4122 | 34.1 |
| 2 | IL1B | rs16944 | 113311337 | A | G | 0.5460 | 34.9 |
| 2 | IRS1 | rs1025333 | 227353768 | A | T | 0.9431 | 7.6 |
| 2 | IRS1 | rs2234931 | 227370996 | A | G | 0.0170 | 6.8 |
| 2 | CXCR7 | rs10183022 | 237146707 | A | G | 0.9946 | 39.3 |
| 2 | CXCR7 | rs9287599 | 237153091 | G | A | 0.0473 | 7.0 |
| 2 | CXCR7 | rs1045879 | 237154642 | T | C | 0.8815 | 27.0 |
| 3 | GHRL | rs35681 | 10304376 | A | G | 0.0306 | 47.7 |
| 3 | GHRL | rs42451 | 10305376 | T | C | 0.5228 | 27.0 |
| 3 | GHRL | rs696217 | 10306456 | T | G | 0.3665 | 8.7 |
| 3 | GHRL | rs34911341 | 10306518 | T | C | 0.3474 | 1.5 |
| 3 | GHRL | rs26802 | 10307364 | G | T | 0.1376 | 31.9 |
| 3 | PPARG | rs1801282 | 12368124 | G | C | 0.3383 | 12.3 |
| 3 | PPARG | rs1800571 | 12397847 |  | C | 0 | 0 |
| 3 | PPARG | rs3856806 | 12450556 | T | C | 0.5304 | 12.1 |
| 3 | KBTBD5 | rs6805421 | 42703147 | A | G | 0.7077 | 35.2 |
| 3 | KBTBD5 | rs123509 | 42708471 | A | G | 0.3597 | 24.1 |
| 3 | KBTBD5 | rs3846062 | 42733667 | T | G | 0.2469 | 39.5 |
| 3 | APEH | rs4855881 | 49690449 | C | T | 0.1277 | 47.4 |
| 3 | APEH | rs2960548 | 49695396 | G | C | 0.2193 | 47.1 |
| 3 | ADIPOQ | rs17300539 | 188042153 | A | G | 0.4412 | 9.5 |
| 3 | ADIPOQ | rs266729 | 188042167 | G | C | 0.1206 | 26.2 |
| 3 | ADIPOQ | rs2241766 | 188053585 | G | T | 0.5963 | 11.5 |
| 3 | ADIPOQ | rs1501299 | 188053816 | A | C | 0.8982 | 29.1 |
| 3 | TFRC | rs2284890 | 197272543 | A | G | 0.3256 | 40.6 |
| 3 | TFRC | rs41301381 | 197282716 |  | C | 0 | 0 |
| 3 | TFRC | rs3817672 | 197285207 | G | A | 0.7495 | 45.9 |
| 3 | TFRC | rs9877119 | 197286762 |  |  | N.A | N.A |
| 4 | ALB | rs3775485 | 74494684 | T | A | 0.0410 | 41.4 |
| 4 | ALB | rs962004 | 74504102 | C | T | 0.0225 | 41.6 |
| 4 | IL8 | rs4073 | 74824887 | A | T | 0.5390 | 46.2 |
| 4 | NFKB1 | rs3774932 | 103643222 | A | G | 0.3705 | 43.8 |
| 4 | NFKB1 | rs1801 | 103720091 | C | G | 0.9769 | 35.8 |
| 4 | UCP1 | rs12502572 | 141704583 | A | G | 0.3028 | 31.0 |
| 4 | UCP1 | rs1800592 | 141713410 | G | A | 0.8819 | 24.3 |
| 4 | TLR2 | rs4696480 | 154826575 | T | A | 0.7890 | 49.9 |
| 4 | TLR2 | rs3804099 | 154844105 | C | T | 0.5947 | 43.9 |
| 4 | TLR2 | rs3804100 | 154844858 | C | T | 0.2870 | 6.2 |
| 4 | TLR2 | rs5743708 | 154845766 | A | G | 0 | 2.7 |
| 5 | IL13 | rs1800925 | 132020707 | T | C | 0.8396 | 18.7 |
| 5 | IL4 | rs2243248 | 132036542 | G | T | 0.0238 | 6.8 |
| 5 | IL4 | rs2070874 | 132037608 | T | C | 0.0788 | 15.8 |
| 5 | CD14 | rs2569190 | 139993099 | A | G | 0.1304 | 44.5 |
| 5 | NR3C1 | rs6195 | 142759509 | G | A | 0.3818 | 3.3 |
| 5 | NR3C1 | rs11749561 | 142771869 | T | C | 0.6635 | 49.1 |
| 5 | ADRB2 | rs1042711 | 148186540 |  |  | N.A | N.A |
| 5 | ADRB2 | rs1042713 | 148186632 | A | G | 0.2396 | 37.9 |
| 5 | ADRB2 | rs1042714 | 148186665 | G | C | 0.2589 | 42.3 |
| 5 | ADRB2 | rs1042717 | 148186838 | A | G | 0.3427 | 19.9 |
| 5 | ADRB2 | rs1800888 | 148187077 | T | C | 0.5638 | 1.3 |
| 5 | ADRB2 | rs1042719 | 148187639 | C | G | 0.0035 | 28.6 |
| 5 | IL12B | rs1368439 | 158674591 | G | T | 0.1290 | 19.6 |
| 6 | LTA | rs909253 | 31648292 | C | T | 0.3938 | 33.3 |
| 6 | TNF | rs1799964 | 31650287 | C | T | 0.9774 | 21.1 |
| 6 | TNF | rs1800629 | 31651010 | A | G | 0.4288 | 16.2 |
| 6 | LY6G5B | rs2142234 | 31747108 | T | C | 0.7024 | 7.2 |
| 6 | LY6G5B | rs9267532 | 31747958 | T | C | 0.9745 | 7.4 |
| 6 | LY6G5B | rs1266076 | 31748497 | C | A | 0.2706 | 36.2 |
| 6 | HSPA1L | rs2227956 | 31886251 |  |  | N.A | N.A |
| 6 | HSPA1B | rs6457452 | 31903529 | T | C | 0.0469 | 6.5 |
| 6 | AGER | rs2070600 | 32259421 | A | G | 0.2786 | 3.9 |
| 6 | CNR1 | rs1049353 | 88910353 | A | G | 0.5675 | 26.5 |
| 6 | FOXO3 | rs9486902 | 108984744 | T | C | 0.0832 | 18.1 |
| 6 | SGK1 | rs1743966 | 134535639 | C | T | 0.8665 | 21.1 |
| 6 | IFNGR1 | rs9389484 | 137571619 |  |  | N.A | N.A |
| 6 | IFNGR1 | rs7749390 | 137582062 | G | A | 0.6075 | 39.5 |
| 7 | IL6 | rs1800795 | 22733169 | C | G | 0.3069 | 42.3 |
| 7 | IL6 | rs2069835 | 22734395 | C | T | 0.0977 | 7.2 |
| 7 | IL6 | rs1554606 | 22735231 | T | G | 0.4882 | 44.0 |
| 7 | IL6 | rs2069845 | 22736673 | G | A | 0.2354 | 44.3 |
| 7 | CAMK2B | rs917791 | 44235309 | C | T | 0.7469 | 41.6 |
| 7 | CAMK2B | rs10441113 | 44255189 | A | G | 0.4894 | 41.0 |
| 7 | CAMK2B | rs4526269 | 44262831 | T | G | 0.5974 | 41.2 |
| 7 | IGFBP3 | rs2453839 | 45920097 | C | T | 0.3267 | 19.2 |
| 7 | IGFBP3 | rs3110697 | 45921554 | A | G | 0.4842 | 40.8 |
| 7 | LEP | rs7799039 | 127666018 | A | G | 0.1478 | 45.1 |
| 8 | LPL | rs1800590 | 19840950 | G | T | 0.5395 | 1.4 |
| 8 | LPL | rs326 | 19863718 | G | A | 0.9355 | 29.7 |
| 8 | LPL | rs328 | 19864003 | G | C | 0.0038 | 10.0 |
| 8 | ADRB3 | rs4994 | 37942954 | C | T | 0.2522 | 6.9 |
| 8 | LY96 | rs6472812 | 75079637 | A | G | 0.8988 | 3.5 |
| 9 | IFNA2 | rs624704 | 21373897 | C | T | 0.5946 | 27.5 |
| 9 | IFNA2 | rs632941 | 21378711 | A | G | 0.9833 | 27.0 |
| 9 | DCTN3 | rs3802427 | 34608640 | A | G | 0.4976 | 17.3 |
| 9 | TLR4 | rs4986790 | 119515122 | G | A | 0.3761 | 4.6 |
| 9 | ZER1 | rs13284665 | 130553190 | G | A | 0.6646 | 12.6 |
| 9 | ZER1 | rs8507 | 130555367 | G | T | 0.1430 | 44.0 |
| 9 | ZER1 | rs4836625 | 130556658 | T | C | 0.7339 | 48.3 |
| 10 | MBL2 | rs1800450 | 54201240 | A | G | 0.0001 | 15.7 |
| 10 | MBL2 | rs5030737 | 54201247 | T | C | <0.0001 | 7.9 |
| 10 | MBL2 | rs7096206 | 54201690 | G | C | 0.1792 | 22.3 |
| 10 | TTC18 | rs4294502 | 74705262 | C | T | 0.7478 | 6.8 |
| 10 | TTC18 | rs3812621 | 74706864 | G | A | 0.4645 | 6.3 |
| 10 | ADRB1 | rs1801253 | 115795045 | G | C | 0.0101 | 29.8 |
| 11 | IGF2 | rs680 | 2110209 | A | G | 0.7149 | 28.3 |
| 11 | NUP160 | rs11039426 | 47819694 | A | G | 0.6973 | 34.5 |
| 11 | ACTN3 | rs1815739 | 66084670 | T | C | 0.5305 | 43.8 |
| 11 | UCP2 | rs660339 | 73366751 | T | C | 0.5325 | 40.4 |
| 11 | UCP2 | rs659366 | 73372401 | T | C | 0.8185 | 36.5 |
| 11 | UCP3 | rs1800849 | 73397812 | T | C | 0.6723 | 25.4 |
| 11 | IL18 | rs360729 | 111522130 | A | T | 0.7971 | 30.6 |
| 11 | IL18 | rs549908 | 111526125 | G | T | 0.9861 | 30.3 |
| 11 | IL18 | rs5744256 | 111528057 | C | T | 0.7047 | 24.0 |
| 11 | IL18 | rs2043055 | 111536833 | G | A | 0.7532 | 36.4 |
| 11 | IL18 | rs187238 | 111540197 | C | G | 0.9032 | 26.8 |
| 11 | IL18 | rs1946519 | 111540716 | A | C | 0.7653 | 40.3 |
| 11 | HYLS1 | rs3088241 | 125268955 | C | G | 0.5639 | 49.5 |
| 11 | HYLS1 | rs549990 | 125271253 | C | A | 0.3540 | 32.2 |
| 11 | HYLS1 | rs622756 | 125271382 | A | C | 0.7626 | 12.6 |
| 11 | HYLS1 | rs547232 | 125278208 | T | C | 0.2080 | 37.5 |
| 12 | ADIPOR2 | rs16928751 | 1760459 | A | G | 0.2649 | 13.7 |
| 12 | ADIPOR2 | rs35854772 | 1763337 | T | G | 0.3277 | 13.8 |
| 12 | TNFRSF1A | rs767455 | 6321205 | C | T | 0.5826 | 42.8 |
| 12 | TNFRSF1A | rs4149570 | 6321850 | T | G | 0.1192 | 40.6 |
| 12 | GNB3 | rs5443 | 6825135 | T | C | 0.9715 | 31.0 |
| 12 | VDR | rs1544410 | 46526101 | A | G | 0.7315 | 41.2 |
| 12 | DCD | rs2029851 | 53327703 | G | A | 0.2402 | 29.2 |
| 12 | IFNG | rs2193049 | 66833188 | C | G | 0.9549 | 27.8 |
| 12 | IFNG | rs2069727 | 66834489 | G | A | 0.1704 | 46.0 |
| 12 | IFNG | rs2430561 | 66838786 | A | T | 0.2565 | 46.4 |
| 12 | IFNG | rs2069709 | 66839969 |  | G | N.A | 0 |
| 12 | IGF1 | rs11111272 | 101351570 | G | C | 0.7214 | 28.5 |
| 12 | IGF1 | rs10735380 | 101368365 | G | A | 0.5285 | 28.5 |
| 13 | FOXO1 | rs2701896 | 40025891 | C | G | 0.8067 | 38.3 |
| 13 | FOXO1 | rs17446593 | 40026084 | G | A | 0.1494 | 17.9 |
| 13 | FARP1 | rs3848017 | 97828589 | C | T | 0.1458 | 25.8 |
| 13 | FARP1 | rs584800 | 97836087 | A | G | 0.4725 | 17.3 |
| 14 | NFKBIA | rs696 | 34940843 | A | G | 0.1690 | 36.2 |
| 14 | AKT1 | rs11555433 | 104312524 |  |  | N.A | N.A |
| 16 | TSC2 | rs7187438 | 2060402 | C | T | 0.8684 | 34.5 |
| 16 | LITAF | rs4280262 | 11554992 | G | A | 0.1265 | 20.5 |
| 16 | MT2A | rs34326929 | 55199886 |  |  | N.A | N.A |
| 16 | MT2A | rs10636 | 55200843 | C | G | 0.7587 | 26.5 |
| 16 | MT1B | rs1875233 | 55244204 | G | A | 0.0897 | 46.3 |
| 16 | FOXC2 | rs34221221 | 85157930 | C | T | 0.1342 | 37.6 |
| 17 | CCL2 | rs1024611 | 29603900 | C | T | 0.2824 | 28.4 |
| 17 | CCL5 | rs2107538 | 31231892 | T | C | 0.1515 | 18.4 |
| 17 | ACE | rs4295 | 58910029 | G | C | 0.3983 | 39.9 |
| 17 | ACE | rs4329 | 58917189 | G | A | 0.1979 | 44.9 |
| 17 | ACE | rs4341 | 58919721 | C | G | 0.1546 | 45.0 |
| 17 | ACE | rs4362 | 58927492 | C | T | 0.1163 | 45.5 |
| 17 | GPS1 | rs4969484 | 77608149 | T | G | 0.4966 | 34.3 |
| 18 | LPIN2 | rs3745012 | 2910287 | T | C | 0.8174 | 25.0 |
| 18 | APCDD1 | rs3748415 | 10461731 | T | C | 0.5587 | 12.0 |
| 18 | MC4R | rs52820871 | 56189811 | G | T | <0.0001 | 1 |
| 18 | MC4R | rs2229616 | 56190255 | A | G | 0.1783 | 2.1 |
| 19 | RETN | rs34124816 | 7639675 | C | A | 0.2203 | 3.6 |
| 19 | RETN | rs1862513 | 7639792 | C | G | 0.5216 | 29.9 |
| 19 | P2RY11 | rs12460842 | 10083194 | A | G | 0.2203 | 3.6 |
| 19 | EIF3G | rs3826785 | 10088148 | T | C | 0.2080 | 11.2 |
| 19 | ICAM1 | rs281432 | 10251657 | G | C | 0.3696 | 46.0 |
| 19 | ICAM1 | rs5498 | 10256682 |  |  | N.A | N.A |
| 19 | GCDH | rs11085824 | 12862546 | G | A | 0.2792 | 37.7 |
| 19 | GCDH | rs9384 | 12871642 | T | G | 0.9174 | 37.3 |
| 19 | GDF15 | rs1058587 | 18360421 | C | G | 0.7051 | 26.6 |
| 19 | TGFB1 | rs1800469 | 46552135 | T | C | 0.6710 | 32.0 |
| 20 | TH1L | rs163781 | 56997159 | G | A | 0.1191 | 45.2 |
| 20 | CTSZ | rs163792 | 57009741 | A | C | 0.0438 | 44.4 |
| 22 | MIF | rs755622 | 22566391 | G | C | 0.9886 | 17.8 |
| 22 | PPARA | rs1800206 | 44992937 | G | C | 0.5375 | 6.2 |

CHR: Chromosome number; BP: SNP base-pair position; A1: Minor allele; A2: Major allele; HWE: Hardy-Weinberg Equilibrium; MAF: minor allele frequency (from main study population). Lines in red denote SNPs removed from final analysis.
